# Impulsivity and Anger Regulation in Adults with Overweight or Obesity and Feeding and Eating Disorders: An Exploratory Cross-Sectional Study

**DOI:** 10.3390/jcm15208156

**Published:** 2026-10-20

**Authors:** Veronica Raspa, Francesca Di Maio, Gianmarco Cinesi, Francesca Scopetta, Patrizia Moretti, Marta Borsellini, Laura Dalla Ragione, Giulia Menculini, Claudia Mazzeschi, Alfonso Tortorella

**Affiliations:** 1 Division of Psychiatry, Department of Neurosciences and Sensory Organs, University Hospital of Perugia, 06129 Perugia, Italy; veronica.raspa1@unipg.it; 2 Section of Psychiatry, Department of Medicine and Surgery, University of Perugia, 06132 Perugia, Italy; francy.dimaio@gmail.com (F.D.M.); gianmarco.cinesi@gmail.com (G.C.); francesca.scopetta@yahoo.it (F.S.); patrizia.moretti@unipg.it (P.M.); alfonso.tortorella@unipg.it (A.T.); 3 Eating Disorders and Obesity Center, Local Health Authority Umbria 1, 06062 Città della Pieve, Italy; marta.borsellini@uslumbria1.it; 4 Food Science and Human Nutrition Unit, University Campus Bio-Medico of Rome, 00128 Rome, Italy; dallaragione@gmail.com; 5 Department of Philosophy, Social Sciences and Education, University of Perugia, 06123 Perugia, Italy; claudia.mazzeschi@unipg.it

**Keywords:** overweight or obesity, feeding and eating disorders, impulsivity, anger regulation

## Abstract

**Background/Objectives**: Feeding and Eating Disorders (FEDs) are common in individuals with overweight or obesity and are associated with greater psychological burden. This study examined whether body-image concerns, impulsivity, and anger regulation distinguish individuals with overweight or obesity with and without a diagnosis of FEDs according to the Diagnostic and Statistical Manual of Mental Disorders (DSM-5-TR). **Methods**: 50 adults with overweight or obesity (76.0% women; mean BMI = 40.23 ± 5.93 kg/m^2^) were recruited from specialist obesity services in Italy. Twenty-one participants (42.0%) had a DSM-5-TR FED diagnosis and 29 did not. FED diagnoses were established using the Structured Clinical Interview for DSM-5 Disorders, Clinician Version (SCID-5-CV). Participants completed the Binge Eating Scale (BES), Body Shape Questionnaire (BSQ), Bulimia Test-Revised (BULIT-R), Barratt Impulsiveness Scale-11 (BIS-11), and State-Trait Anger Expression Inventory-2 (STAXI-2). Group differences and an exploratory logistic regression analysis were performed. **Results**: Compared with participants without FED, those with FED showed significantly higher eating-disorder psychopathology. Body-image concerns were higher in the FED group but did not reach statistical significance. Participants with FED showed higher motor impulsivity and poorer anger regulation. Motor impulsivity was independently associated with FED diagnosis (OR = 1.474, 95% CI = 1.084–2.003, *p* = 0.013) and remained significant after adjusting for recruitment site. An association between lower anger control and FED diagnosis was also observed but did not remain significant after this adjustment. **Conclusions:** Adults with overweight or obesity and FED present psychological characteristics extending beyond eating-disorder psychopathology; motor impulsivity emerged as the most robust transdiagnostic correlate of FED status, whereas the association with anger regulation was less consistent and did not survive adjustment for recruitment site. These findings highlight the importance of multidimensional psychological assessment and should be confirmed in larger, prospective, adequately powered studies.

## 1. Introduction

Obesity is a chronic, multifactorial disease characterized by excessive fat accumulation that can impair health, while overweight also carries health risks, with both conditions arising from the interaction of biological, behavioral, social, environmental, and psychological factors [1]. Traditionally, overweight and obesity have been defined according to the degree of excess adiposity. However, recent evidence has identified these as a heterogeneous conditions whose clinical significance depends not only on the amount of excess adiposity but also on the extent to which adiposity compromises health functioning [2]. Accordingly, current scientific consensus supports a multidimensional assessment of overweight and obesity to better characterize disease severity and guide clinical management [2,3]. Within this multidimensional framework, psychological and psychiatric assessment represents an essential component of the clinical evaluation of overweight or obesity. Feeding and Eating Disorders (FEDs), as defined in the Diagnostic and Statistical Manual of Mental Disorders (DSM-5-TR), comprise a group of disorders characterized by persistent disturbances in eating behaviors and related thoughts and emotions that result in significant physical or psychosocial impairment [4]. FEDs are highly prevalent among individuals with overweight or obesity and are also associated with increased psychiatric comorbidity, higher levels of psychological distress, poorer psychosocial functioning, and less favorable treatment outcomes compared with overweight or obesity alone [5,6]. Recent evidence further confirms the high prevalence of FEDs among individuals with overweight or obesity, highlighting the importance of their systematic identification [7]. Beyond the assessment of eating-disorder psychopathology, body image concerns represent another key psychological dimension in individuals with overweight or obesity. Body image is a multidimensional construct involving cognitive, affective, perceptual, and behavioral components related to body shape and weight [8]. Body-image concerns are common among individuals with overweight or obesity and have been consistently associated with psychological distress and eating-disorder psychopathology [9,10]. The multidimensional conceptualization of overweight or obesity has also shifted attention toward psychological factors, which are increasingly recognized as important contributors to its clinical heterogeneity [2,3]. Rather than being exclusively linked to the presence or absence of a specific psychiatric diagnosis, these psychological dimensions may vary in severity across individuals with overweight or obesity. Among these dimensions, impulsivity has received attention because of its potential role in self-regulation and eating behavior. Impulsivity is defined as a multifaceted and transdiagnostic neurobehavioral trait that involves acting with a predisposition toward rapid, unplanned, and inadequately thought-out behavior without fully considering the potential consequences of such behavior [11]. Elevated impulsivity has been associated with overeating and greater vulnerability to maladaptive eating behaviors [12]. Higher levels of impulsivity have also been reported in individuals with FEDs, supporting the view that impulsivity may represent a transdiagnostic psychological process underlying both overweight or obesity and FED psychopathology [6]. Anger-related dimensions have received increasing attention in overweight or obesity and eating-disorder research, particularly within models emphasizing emotion dysregulation. Indeed, eating and loss-of-control eating have been conceptualized as maladaptive attempts to regulate negative affective states. According to emotion-regulation models of binge eating, negative emotions may precede binge-eating episodes, while food intake may temporarily reduce emotional distress, thereby reinforcing the behavior over time [13,14]. In this framework, anger may represent a particularly relevant, yet less frequently investigated, emotional state. The clinical significance of anger in individuals with overweight or obesity and binge eating may depend less on the intensity of anger per se than on how anger is expressed, inhibited, or controlled. Indeed, previous research has supported the broader relevance of emotion regulation and inhibitory-control processes in FEDs and overweight or obesity, suggesting that difficulties in regulating affective states may contribute to disordered eating through emotional and behavioral pathways [14,15,16]. Eating-disorder psychopathology, body image concerns, impulsivity, and anger-related dimensions have been investigated in adults with overweight or obesity, but some points need further clarification. First, previous studies have investigated dimensional constructs of eating-disorder psychopathology rather than in those with and without a DSM-5-TR diagnosis of FEDs. Second, little is known about whether the presence of a FED diagnosis is associated with transdiagnostic psychological dimensions extending beyond eating symptoms. Another matter to be considered is impulsivity. Whilst impulsivity has been associated with binge eating and reward-related eating behaviors in many studies, theoretical models understand impulsivity as a multidimensional construct [11,15,17]. Therefore, the question remains whether certain facets of impulsivity (as opposed to impulsivity as a unidimensional trait) are related to the occurrence of a FED. Finally, anger-related dimensions remain underexplored in this field. Clarifying this issue may help shift the focus from anger proneness to anger-regulation abilities as clinically relevant correlates of FED status. The primary aim of this exploratory study was to compare adults with overweight or obesity with and without a DSM-5-TR diagnosis of FEDs on multiple psychological domains, including eating-disorder psychopathology, body image, impulsivity, self-control, and anger-related constructs.

**Aim** **1.** *To explore differences in eating-disorder psychopathology, body-image concerns, impulsivity, and anger-related measures between participants with and without a DSM-5-TR diagnosis of a FED.*

**Expected** **Pattern 1.** *Consistent with previous evidence indicating that individuals with overweight or obesity and comorbid FEDs show more severe psychological distress, participants with a diagnosis of a FED were expected to report higher levels of eating-disorder psychopathology, impulsivity, and body-image difficulties compared to those without a FED diagnosis [5,6].*

**Aim** **2.** *To identify which transdiagnostic psychopathological dimensions showing significant between-group differences were independently associated with a diagnosis of FED.*

**Expected** **Pattern 2. ** *Based on previous evidence and the transdiagnostic framework adopted in the present study, measures of eating-disorder psychopathology, body image, and impulsivity were expected to emerge as independent exploratory correlates of a FED diagnosis [5,6,12]. Given the exploratory nature of the evidence, no specific hypothesis was formulated regarding anger-related dimensions.*

## 2. Materials and Methods

### 2.1. Participants

**Figure 1:**
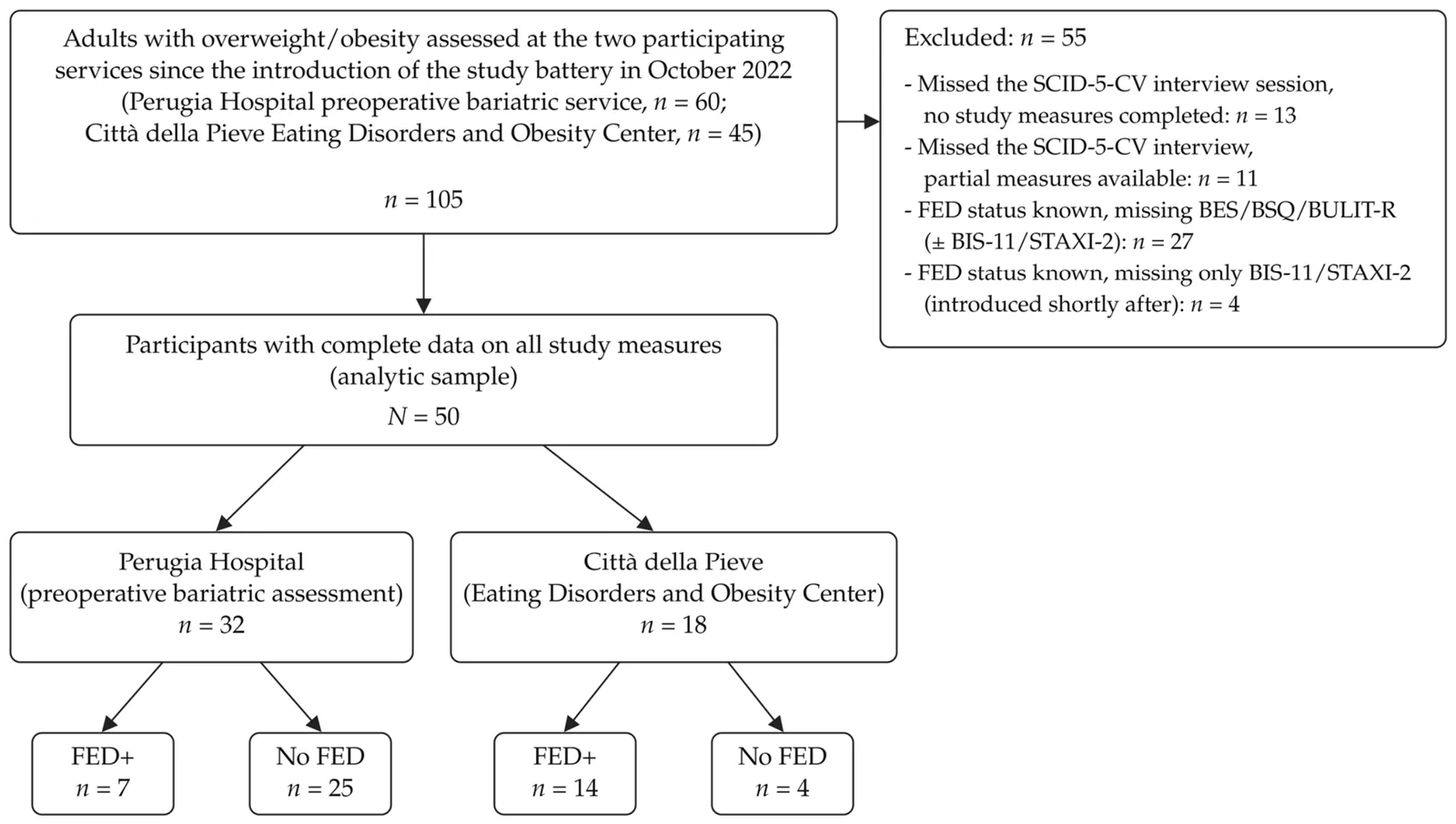
Flow diagram of participant selection.

The sample consisted of 50 individuals with overweight or obesity recruited from clinical services in the Umbria Region (Italy): the Psychiatric Outpatient Service of Perugia Hospital, where 32 participants underwent the standard preoperative assessment for bariatric surgery, and the Eating Disorders and Obesity Center of Città della Pieve, where 18 patients were evaluated as part of a specialized multidisciplinary treatment program. Biological sex was recorded as documented in the clinical file at the time of assessment. Following the introduction, in October 2022, of a structured psychological assessment battery for FED-related psychopathology (including the SCID-5-CV interview, the BES, BSQ, BULIT-R, BIS-11, and STAXI-2) at both participating services, 105 adults with overweight or obesity were assessed (Perugia Hospital, *n* = 60; Città della Pieve, *n* = 45). Of these, 55 were excluded from the present analysis because of incomplete data (Figure 1), based on a missing at random (MAR) pattern: 13 participants missed the scheduled interview session and did not complete the SCID-5-CV, and thus had no study measures available; 11 had a partially completed questionnaire battery but no SCID-5-CV-based FED classification, for the same reason; 27 had a confirmed FED status but incomplete data on the BES, BSQ, and/or BULIT-R; and 4 had complete eating-disorder-specific measures but missing BIS-11 and/or STAXI-2 data, reflecting the brief interval between the introduction of the eating-disorder measures and that of the impulsivity and anger-regulation measures within the same battery. Only participants with complete data on all questionnaires and a SCID-5-CV-based FED classification were retained in the final analytic sample (N = 50; see Figure 1 and Section 2.5). Participants were assessed between 2022 and 2024 in the pre-bariatric surgery setting and between 2025 and 2026 in the specialist eating-disorder and obesity center, with no temporal overlap between the two recruitment periods. Because the study was retrospective and based on the availability of complete questionnaire data, recruitment was not strictly consecutive within either setting.

### 2.2. Procedure

Data were retrospectively extracted from clinical databases maintained by two services in the Umbria Region: the Psychiatry Outpatient Service of Perugia Hospital and the Eating Disorders and Obesity Center of Città della Pieve. At Perugia Hospital, participants were assessed as part of the standard preoperative evaluation for bariatric surgery. At the Città della Pieve center, participants were assessed within a specialized multidisciplinary treatment pathway for eating disorders and overweight or obesity. Both services used the same standardized psychological assessments. The psychiatric assessment included a clinical interview, self-report questionnaires, and diagnostic evaluation using the Structured Clinical Interview for DSM-5 Disorders, Clinician Version (SCID-5-CV) [18]. Current and lifetime psychiatric diagnoses, including FED diagnoses, were assessed at the time of the clinical evaluation using the SCID-5-CV. Since we considered both current and lifetime comorbidities, the co-existence of two distinct FEDs or other psychiatric disorders was also registered. Interviews were administered by third- and fourth-year psychiatry residents trained in the use of the instrument during the first two years of their residency by attending mandatory courses for the use of psychometric tests. Personality disorders were additionally assessed using the Structured Clinical Interview for DSM-5 Personality Disorders (SCID-5-PD/SPQ) [19]. The same team of psychiatry residents, trained as described above, conducted diagnostic interviews across both recruitment centers, ensuring a standardized diagnostic procedure across the two clinical settings. FED diagnoses were established exclusively on the basis of DSM-5-TR criteria as assessed through the SCID-5-CV and overall clinical judgment; self-report questionnaire scores (BES, BSQ, BULIT-R, BIS-11, STAXI-2) were not used as formal diagnostic criteria and did not directly determine the diagnostic decision. However, interviewers were not blinded to questionnaire responses, and no formal inter-rater reliability assessment was performed. Questionnaires were completed under clinician supervision. Inclusion criteria were age ≥ 18 years, overweight or obesity, adequate knowledge of Italian, completion of the assessments, and written informed consent for the clinical and research use of anonymized data. The study was conducted in accordance with the ethical principles of the Declaration of Helsinki. All participants provided written informed consent for the clinical and research use of their anonymized data prior to participation. The research protocol was approved by the Local Ethics Committee of the Umbria Region (CER Umbria; protocol No. 23357121/NCOV, 24 November 2021).

### 2.3. Measures

The Binge Eating Scale (BES) [20]: a 16-item self-report questionnaire that measures the severity of binge-eating symptomatology. The scale measures behavioral manifestations and cognitive and emotional aspects of binge eating. Higher total scores indicate greater severity of binge-eating symptoms. The Italian version has demonstrated good psychometric properties in individuals with overweight and obesity, supporting a unidimensional factor structure and adequate reliability and validity [20]. 

The Body Shape Questionnaire (BSQ) [21]: a 34-item self-report questionnaire designed to assess concerns related to body shape and weight. The measure evaluates body dissatisfaction, fear of weight gain, and body-image-related distress. Higher scores indicate greater body shape concerns. The Italian version has demonstrated excellent psychometric properties, supporting a unidimensional factor structure, excellent internal consistency, and good test–retest reliability in clinical and non-clinical samples [22]. 

The Bulimia Test-Revised (BULIT-R) [23]: a 28-item self-report questionnaire, rated on a 5-point Likert scale, designed to assess the presence and severity of bulimic symptomatology consistent with diagnostic criteria for bulimia nervosa. The instrument evaluates the frequency of binge-eating episodes, subjective loss of control while eating, use of compensatory behaviors (e.g., self-induced vomiting, laxative misuse, fasting, excessive exercise), and preoccupation with body weight and shape. Total scores range from 28 to 140, with higher scores indicating greater severity of bulimic symptomatology. No formal Italian validation of the BULIT-R was identified in the literature; its use in the present sample was supported by strong convergent validity with the BES (*r* = 0.675, *p* < 0.001) and the BSQ (*r* = 0.498, *p* < 0.001), both validated Italian instruments assessing overlapping eating-disorder constructs. 

The Barratt Impulsiveness Scale-11 (BIS-11) [24]: a 30-item self-report measure of trait impulsivity, rated on a 4-point Likert scale. The questionnaire comprises three second-order dimensions: Attentional Impulsiveness, Motor Impulsiveness, and Non-planning Impulsiveness; derived from six first-order factors (Attention, Cognitive Instability, Motor, Perseverance, Self-Control, and Cognitive Complexity). Between-group comparisons (Section 3.2) were conducted on the BIS-11 total score, the three second-order dimensions (Attentional, Motor, and Non-planning Impulsiveness), and the Self-Control first-order factor. Based on these univariate results, only Motor and Non-planning Impulsiveness—which showed statistically significant between-group differences without substantial overlap with the total score—were retained as independent variables in the exploratory logistic regression (Section 2.4); the BIS-11 total score was excluded due to multicollinearity with its subscales, and Attentional Impulsiveness (*p* = 0.073) and Self-Control (*p* = 0.224) did not differ significantly between groups and were therefore not entered. Because impaired self-control is theoretically and clinically central to loss-of-control eating and binge-eating behavior, the Self-Control first-order factor—one of the two first-order components underlying Non-planning Impulsiveness—was additionally examined on its own, to determine whether this specific facet showed an association with FED status beyond the broader second-order dimension. The Self-Control factor assesses planning, deliberation, and self-regulatory abilities; consistent with the BIS-11 scoring system, higher scores reflect poorer self-control, rather than better self-control. The Italian version of the scale has shown satisfactory psychometric properties [25]. 

State-Trait Anger Expression Inventory (STAXI-2) [26]: a 57-item self-report questionnaire assessing anger experience, expression, and regulation through six scales: State Anger, Trait Anger, Anger Expression-Out, Anger Expression-In, Anger Control-Out, and Anger Control-In. Items are rated on a 4-point Likert scale, with higher scores indicating greater levels of the corresponding dimension. The full STAXI-2 was administered, yielding the six above-mentioned scales, which were examined in the univariate group comparisons (Section 3.2). Scale scores were expressed as T scores, obtained with the same conversion at both recruitment centers. Anger Control-Out and Anger Control-In were additionally combined into a study-specific Anger Control Index (see Section 2.4) for use in the multivariable model.

### 2.4. Statistical Analysis

Analyses were conducted on participants with complete questionnaire data (see Section 2.5 for missing data). The multivariable analyses were based on all 50 participants, who had complete data on all predictors. Occasional missing values for age (*n* = 2), BMI (*n* = 1), and STAXI-2 Trait Anger-Temperament (*n* = 1) were handled pairwise. Descriptive statistics were computed for all socio-demographic, clinical and psychological variables. Continuous variables are reported as means and standard deviations (M, SD), whereas categorical variables are presented as frequencies and percentages. Group differences between participants with and without a FED diagnosis were examined using independent-samples *t*-tests. Homogeneity of variance was assessed using Levene’s test. Candidate predictors for the exploratory logistic regression were restricted a priori to two transdiagnostic psychological domains with an established conceptual link to eating-disorder psychopathology in the literature—impulsivity and anger regulation—whereas eating-specific symptom scales were excluded a priori regardless of their univariate significance, to avoid criterion overlap with the FED diagnosis. Within these two domains, the specific subscales showing significant univariate group differences were retained as candidate predictors; given the small sample and limited number of events, this two-step selection strategy is itself exploratory, and the resulting model should be interpreted as hypothesis-generating instead of confirmatory. The Anger Control-Out and Anger Control-In subscales were averaged into a composite score because of their extremely high correlation in the present sample (*r* = 0.953). We acknowledge that a high correlation alone does not establish that the two subscales form a valid unidimensional construct, and that this composite is not part of standard STAXI-2 scoring. To avoid relying on this assumption, we repeated the multivariable analysis entering Anger Control-Out and Anger Control-In separately (each with Motor and Non-planning Impulsivity; Table S3.3), obtaining virtually identical results (OR = 0.922, *p* = 0.045 and OR = 0.922, *p* = 0.044, respectively) to those obtained with the composite (OR = 0.920, *p* = 0.042). The substantive conclusion regarding anger control is therefore not dependent on treating the two subscales as a single dimension, and both the composite and the single-subscale models are reported for transparency. Trait Anger and Anger Expression-Out/In, although significantly associated with FED status in univariate analyses, were not entered into any of these models together with the anger-control subscales because of their very high intercorrelation with one another (*r* = 0.80–0.86) and substantial overlap with Anger Control-Out/-In themselves (*r* = 0.65–0.71), which would have introduced severe multicollinearity in a model already limited by sample size. The anger-control subscales—whether combined or entered individually—were retained as the anger-related predictors because they showed comparatively greater independence from the impulsivity measures (*r* = −0.36 to −0.59) and were conceptually most consistent with the study’s focus on anger-regulation capacity rather than anger intensity or expression per se. The dependent variable was the presence of a FED diagnosis. The selected impulsivity and anger-control scores were entered as predictors. Model fit was evaluated using the Hosmer–Lemeshow goodness-of-fit test and Nagelkerke’s R^2^. Odds ratios (ORs) and 95% confidence intervals (CIs) were calculated for all independent variables. Given the exploratory nature of the study and the number of comparisons performed, Benjamini–Hochberg false discovery rate (FDR) correction was applied to the sixteen group comparisons, and effect sizes are reported as Hedges’ g with 95% confidence intervals and not Cohen’s d, given the small subgroup sizes. When Welch’s *t*-test was used because of unequal variances, Hedges’ g and its confidence interval were nonetheless computed using the pooled standard deviation, consistent with standard effect-size conventions; this approach does not incorporate the variance-weighting used by Welch’s test itself and may therefore slightly understate the standard error in these specific comparisons. Normality was assessed within each group using the Shapiro–Wilk test, and influential observations were screened using Tukey’s 1.5 × interquartile range rule; Mann–Whitney U tests were performed as a non-parametric sensitivity analysis for all sixteen comparisons. For the multivariable logistic regression, given the limited number of FED events (*n* = 21) relative to the number of predictors, we additionally re-estimated the model using Firth’s penalized (bias-reduced) logistic regression, performed bootstrap-based internal validation (2000 resamples) to obtain an optimism-corrected estimate of model discrimination, and report standardized odds ratios (per 1-SD increment) alongside the original estimates. Because Anger Control-Out and Anger Control-In were highly collinear (*r* = 0.953; VIF > 11 when entered jointly), sensitivity models entering each subscale separately were also performed. All statistical tests were two-tailed, and statistical significance was set at *p* < 0.05. Analyses were conducted using IBM SPSS Statistics v. 26.0 (IBM Corp., Armonk, NY, USA).

### 2.5. Missing Data

Missing data were primarily related to three identifiable mechanisms: (a) discontinuation of the assessment pathway before the SCID-5-CV interview could be administered (*n* = 24), because participants missed the scheduled diagnostic session; (b) a confirmed FED classification with incomplete data on the eating-disorder-specific questionnaires (BES, BSQ, and/or BULIT-R; *n* = 27); and (c) the brief delay between the introduction of the eating-disorder-specific questionnaires and that of the BIS-11 and STAXI-2 within the same battery (*n* = 4). Among retained participants, age was missing for 2 (both in the FED group), BMI for 1 (No FED), and Trait Anger-Temperament for 1 (No FED). To assess the potential for selection bias, participants retained in the analytic sample (N = 50) were compared with those excluded for incomplete data (*n* = 55) on the sociodemographic and clinical variables available for both groups in the source clinical records. No statistically significant differences emerged in age (46.8 ± 13.8 vs. 47.0 ± 14.0 years, *p* = 0.934), BMI (40.2 ± 5.9 vs. 42.6 ± 8.3 kg/m^2^, *p* = 0.125), sex distribution (76.0% vs. 78.2% female, χ^2^ = 0.00, *p* = 0.973), or recruitment site (χ^2^ = 1.34, *p* = 0.248). Because a formal FED classification could not be established for most excluded participants (*n* = 24/55), a direct comparison on FED-related variables was not feasible; this is acknowledged as a limitation in Section 4.

## 3. Results

### 3.1. Sample Description

Participants in the sample had a mean age of 46.77 years (SD = 13.82; range 19–78 years) and a mean BMI of 40.23 kg/m^2^ (SD = 5.93; range 29–53 kg/m^2^). Forty-nine participants (98.0%) met BMI criteria for obesity, whereas one participant (2.0%) was overweight. The majority were female (76.0%, *n* = 38). According to the SCID-5-CV, 21 participants (42.0%) fulfilled the DSM-5-TR criteria of a FED, while 29 (58.0%) did not receive a diagnosis of FED. The most represented FED diagnosis was Unspecified Feeding or Eating Disorder (UFED) (*n* = 11, 22%), followed by Bulimia Nervosa (BN) (*n* = 5, 10%), Binge Eating Disorder (BED) (*n* = 3, 6%), and Other Specified Feeding and Eating Disorder (OSFED) (*n* = 2, 4%). None of the included patients showed comorbidity between two or more FEDs. As for other psychiatric comorbidities, these were present in 6 (12%) patients and were distributed as follows: mood disorders (*n* = 2, 4%), personality disorders (*n* = 2, 4%), anxiety disorders (*n* = 1, 2%), and alcohol-related disorders (*n* = 1, 2%). Substantial variability was observed across measures of eating-disorder psychopathology, body image concerns, impulsivity and anger-related dimensions. Descriptive statistics for demographic, clinical, and psychological variables are presented in Table 1.

### 3.2. Group Comparisons

Participants with and without a FED diagnosis did not differ significantly in age (M = 45.26 vs. 47.76 years, SD = 15.54 vs. 12.75; t(46) = 0.61, *p* = 0.546), BMI (M = 40.65 vs. 39.91 kg/m^2^, SD = 7.15 vs. 4.94; Welch’s t(33.70) = −0.41, *p* = 0.687), or sex distribution (χ^2^(1) = 0.00, *p* = 1.00). In contrast, FED prevalence differed markedly by recruitment center, with 7/32 (21.9%) participants assessed at the pre-bariatric service and 14/18 (77.8%) participants assessed at the Eating Disorders and Obesity Center meeting FED criteria (χ^2^(1) = 14.78, *p* < 0.001). As for psychopathological characteristics, participants with a FED diagnosis reported significantly higher levels of binge-eating symptomatology, as assessed by the BES, t(48) = −4.63, *p* < 0.001, as well as bulimic symptomatology, as assessed by the BULIT-R, t(48) = −4.04, *p* < 0.001. Large effect sizes were found for both measures. No statistically significant between-group differences emerged for body image (BSQ). Regarding impulsivity, participants with a FED diagnosis showed significantly higher total scores on the BIS-11, t(48) = −3.49, *p* = 0.001. More specifically, they reported significantly higher levels of motor impulsivity, Welch’s t(27.12) = −4.71, *p* < 0.001, and non−planning impulsivity, t(48) = −2.04, *p* = 0.047. Participants with FEDs had significantly lower Anger Control-Out and Anger Control-In scores than those without FED, with large between-group effect sizes. Descriptive statistics, tests of significance, and effect sizes are displayed in Table 2.

After Benjamini–Hochberg correction for all sixteen comparisons in Table 2, differences remained statistically significant for BES, BULIT-R, BIS-11 total score, Motor Impulsivity, Trait Anger, Trait Anger-Temperament, Trait Anger-Reaction, Anger Expression-Out, Anger Expression-In, Anger Control-Out, and Anger Control-In (all FDR-adjusted *p* < 0.04), whereas Non-planning Impulsivity (unadjusted *p* = 0.047, FDR-adjusted *p* = 0.062) did not survive correction, consistent with BSQ, Attentional Impulsivity, Self-Control, and State Anger, none of which reached significance before or after correction.

### 3.3. Logistic Regression

To explore transdiagnostic correlates of FED status, motor impulsivity, non-planning impulsivity, and anger-control indicators—the subscales showing significant univariate group differences within the a priori conceptual domains of impulsivity and anger regulation—were entered into an exploratory binary logistic regression model; this model-building strategy, and the resulting odds ratios, should not be interpreted as confirmatory evidence of independent predictive effects. The dependent variable was the presence of a FED diagnosis. As already stated, scales assessing FED-specific psychopathology were not entered in the model due to possible overlap with the dependent variable construct. Given the extremely high correlation between STAXI-2 Anger Control-Out and Anger Control-In scores in the present sample (r = 0.953) and the presence of multicollinearity between the two subscales, these were averaged into a study-specific STAXI-2 Anger Control Index. This composite was used to reduce redundancy and model complexity in the exploratory logistic regression and was interpreted as an overall index of anger-regulation/control abilities. We also entered motor and non-planning impulsivity as independent variables in the model, while BIS-11 total score was excluded due to the violation of multicollinearity assumption. The overall model was statistically significant, χ^2^(3) = 26.43, *p* < 0.001, indicating that the included independent variables reliably distinguished participants with a FED diagnosis from those without a FED diagnosis. The model had good explanatory power (Nagelkerke R^2^ = 0.552), and the Hosmer–Lemeshow test showed no evidence of poor calibration (χ^2^ = 4.51, *p* = 0.808); however, with only 50 observations, this test has limited power to detect miscalibration, and this result should not be interpreted as strong evidence of good calibration. Overall classification accuracy was 78.0%. Among the variables included in the model, motor impulsivity and anger control emerged as significant independent correlates of a FED diagnosis. Higher levels of motor impulsivity were associated with greater odds of a FED diagnosis (OR = 1.474, 95% CI [1.084, 2.003], *p* = 0.013), while anger control was negatively associated with the presence of a FED diagnosis (OR = 0.920, 95% CI [0.848, 0.997], *p* = 0.042). The results are reported in Table 3. To evaluate whether recruitment site accounted for the independent correlates identified in the primary logistic regression model, we repeated the analysis including recruitment site as an additional covariate. The overall model remained statistically significant, χ^2^(3) = 26.21, *p* < 0.001, Nagelkerke R^2^ = 0.549. Motor impulsivity remained significantly associated with FED status (OR = 1.389, 95% CI [1.086, 1.778], *p* = 0.009), whereas the association between the STAXI-2 Anger Control Index and FED status was no longer statistically significant (OR = 0.968, 95% CI [0.813, 1.154], *p* = 0.719). The estimate for recruitment site itself was highly imprecise (OR = 2.864, 95% CI [0.073, 112.704], *p* = 0.574), reflecting the small sample size and the substantial imbalance in FED prevalence and psychological profile across recruitment settings, which were also entirely non-overlapping in time.

To address concerns about overfitting given the limited number of FED events relative to the number of predictors, the multivariable model was additionally re-estimated using Firth’s penalized logistic regression (Table S3.1). Motor impulsivity remained significant (OR = 1.38, 95% CI [1.05, 1.81], *p* = 0.020), Non-planning impulsivity remained non-significant (OR = 0.94, 95% CI [0.77, 1.14], *p* = 0.534), and the association with the Anger Control Index was attenuated below conventional significance (OR = 0.93, 95% CI [0.86, 1.00], *p* = 0.059). Model diagnostics for the primary (MLE) model did not indicate relevant violations of the assumptions underlying binary logistic regression: variance inflation factors were low for all three predictors (motor impulsivity = 1.79, non-planning impulsivity = 1.33, Anger Control Index = 1.56), a Box-Tidwell test provided no evidence against linearity of the logit for any predictor (all interaction *p* > 0.23), and neither Cook’s distance (maximum = 0.29) nor leverage (maximum = 0.224) indicated that any single observation disproportionately influenced the results, although five and seven observations respectively exceeded conventional influence thresholds (4/n and twice the mean leverage), consistent with the modest sample size rather than with undue leverage by specific cases. Bootstrap-based internal validation (2000 resamples) indicated modest optimism in model discrimination (apparent AUC = 0.877, 95% CI [0.757, 0.962]; optimism-corrected AUC = 0.849) and confirmed the differential stability of the three predictors: the bootstrap-derived 95% confidence interval for motor impulsivity excluded 1 (OR 95% CI [1.196, 3.436]), whereas those for non-planning impulsivity ([0.612, 1.179]) and the Anger Control Index ([0.783, 1.007]) each included or closely approached 1, indicating comparatively less stable coefficient estimates. As the aim of the analysis was to identify exploratory psychological correlates of FED status, the classification accuracy reported in earlier analyses is not emphasized in the present results. For completeness, the corresponding probability cutoff, sensitivity, specificity, and predictive values are reported in the note to Table 3.

### 3.4. Recruitment Site: Descriptive Comparison and Sensitivity Analysis

FED status differed by recruitment site: 7/32 participants (21.9%) met FED criteria in the pre-bariatric setting compared with 14/18 (77.8%) in the specialist center, χ^2^(1) = 14.78, *p* < 0.001. The two sites did not differ in age (46.75 ± 10.66 vs. 46.81 ± 19.06 years, Welch t(19.83) = -0.01, *p* = 0.990) or sex distribution (75.0% vs. 77.8% female, χ^2^ = 0.05, *p* = 0.825), but differed significantly in BMI (41.70 ± 5.14 vs. 37.46 ± 6.46 kg/m^2^, t(47) = 2.51, *p* = 0.016, Hedges’ g = 0.74) and on several clinical measures, including eating-disorder psychopathology, impulsivity, and anger-related scores (Table 4).

## 4. Discussion

The present pilot exploratory cross-sectional study investigated psychological correlates of FEDs, diagnoses according to DSM-5-TR criteria, in a clinical sample of adults with overweight or obesity. Participants with FEDs showed higher levels of eating-disorder psychopathology, as reflected by significantly higher BES and BULIT-R scores. Moreover, a FED diagnosis was associated with higher trait impulsivity, particularly motor impulsivity, and with lower anger-control abilities. In an exploratory logistic regression focused on transdiagnostic dimensions, motor impulsivity and the STAXI-2 Anger Control Index emerged as independent correlates of FED status, whereas non-planning impulsivity did not retain statistical significance. The presence of higher eating psychopathology in FED subjects was expected and supports the clinical validity of the diagnostic grouping. However, the association between a FED diagnosis and eating-specific scales should be interpreted with caution, as these measures are closely related to the diagnostic construct itself. For this reason, the exploratory regression model deliberately focused on transdiagnostic variables rather than on eating-disorder symptom scales. Body image concerns showed a moderate between-group effect but did not reach statistical significance. However, this result deserves consideration. Body image concerns are highly prevalent among individuals with overweight or obesity [9,10]. Therefore, high BSQ scores may characterize individuals with overweight or obesity more broadly than specifically those with FED. In this sense, the non-significant BSQ difference in our sample may suggest that body-image concerns are clinically relevant across individuals with overweight or obesity, whereas impulsivity and anger-regulation difficulties may better differentiate participants with comorbid FEDs. It is also worth considering that the predominantly female composition of the sample may have contributed to this pattern: because body-image concerns tend to be highly prevalent among women with overweight or obesity regardless of comorbid FED status, a female-predominant sample may be less able to detect FED-specific differences in body image, whereas different patterns might emerge in more sex-balanced samples. This possibility could not be formally tested, given the limited number of male participants in the present sample, and thus represents an area for future investigation. This interpretation is consistent with evidence showing that body image concerns represent one component of a broader psychological profile and not a specific indicator of a FED diagnosis [9]. A central finding of this study concerns impulsivity. Participants with FEDs showed higher BIS-11 total scores, with particularly marked differences in motor and non-planning impulsivity. Moreover, motor impulsivity remained independently associated with FED diagnosis in the exploratory logistic regression. This supports the view that impulsivity should not be treated as a unitary construct in overweight or obesity and eating-disorder research [15,17]. Motor impulsivity, defined as the tendency to act rapidly without adequate forethought, could be relevant to loss-of-control eating, binge episodes, and reward-driven eating behaviors [27]. Overall, the behavioral/acting component of impulsivity may be more closely related to FED status than the cognitive/planning component. This is in line with evidence indicating that specific BIS-11 facets, especially motor impulsivity, are more strongly associated with binge eating and overweight or obesity-related eating dysregulation than other impulsivity dimensions [15,16,28]. The second key finding concerns anger regulation. Beyond anger control, participants with FEDs also reported lower Trait Anger and lower Anger Expression, both outward and inward, compared with those without FED. As with the anger-control association discussed below, these univariate differences should be interpreted with caution given the marked confounding between recruitment site, calendar period, and STAXI-2 scores in this sample; unlike Anger Control, they were not entered into the multivariable or site-adjusted models and are reported here descriptively. The pattern underlined by our results suggests a broader anger profile characterized not by heightened anger proneness, but by a generally lower propensity to experience and express anger combined, paradoxically, with a reduced capacity to regulate it when it does arise. This configuration may be consistent with an inhibited or restrictive emotional style, in which anger is less frequently experienced or expressed but poorly modulated when present—a pattern that warrants further investigation using emotion-regulation frameworks specific to eating psychopathology. Given the strong intercorrelation among Trait Anger, Anger Expression, and Anger Control scores in the present sample, the exploratory regression model focused on the anger-control subscales—examined both as a combined index and, in sensitivity analyses, as separate Anger Control-Out and Anger Control-In scores—as the anger-related correlate of FED status, given their comparatively lower collinearity with the impulsivity measures relative to Trait Anger or Anger Expression. This finding is also consistent with emotion-regulation models of binge eating, according to which binge episodes may function as maladaptive strategies to modulate negative affective states [13,14]. In particular, anger may represent a relevant affective state in binge-eating psychopathology, especially when considered in terms of regulation rather than anger intensity alone [14,16]. To note, the association between lower anger control and FED status did not remain significant after adjusting for recruitment site, and should therefore be interpreted with substantial caution. Recruitment site differed markedly in FED prevalence and was confounded with the recruitment period, making it statistically impossible, in the present sample, to disentangle a genuine FED-specific effect on anger regulation from a site- or cohort-level difference. Motor impulsivity, by contrast, remained significantly associated with FED status after this adjustment, supporting it as the more robust transdiagnostic correlate identified in this study. Given the very small subsample sizes once stratified by both site and FED status, the site-adjusted model itself should be regarded as exploratory and imprecise as reflected in the wide confidence interval for the site estimate. This limitation is central to the interpretation of the anger-related findings and should guide future research toward multi-site designs with balanced FED distribution across settings and concurrent recruitment periods. Taken together, motor impulsivity was the only correlate that remained significant across every robustness check performed, including FDR correction, non-parametric testing, Firth’s penalized regression, standardization, and adjustment for recruitment site. Non-planning impulsivity and the anger-control composite, by contrast, did not survive one or more of these checks, indicating that these associations should be considered preliminary and more sensitive to analytic choices in a sample of this size. This pattern strengthens confidence in motor impulsivity as the most robust transdiagnostic correlate identified in this exploratory study, while anger regulation warrants replication before being considered an independent correlate of FED status. Given the cumulative uncertainty surrounding the anger-control finding—its attenuation after adjustment for recruitment site, its reduced significance under Firth’s bias-corrected estimation, and the conceptual limitations of combining Anger Control-Out and Anger Control-In into a single score—we treat this association as a secondary observation. From a clinical perspective, and bearing in mind the preliminary nature of the anger-control finding, we should nonetheless underline that the co-occurrence of higher motor impulsivity and lower anger control, if confirmed in future studies, may identify a subgroup of individuals with overweight or obesity with difficulties in behavioral inhibition and emotional self-regulation. Indeed, individuals who tend to act rapidly under emotional pressure and have fewer anger-regulation resources could be considered as more vulnerable to dysregulated eating episodes, interpersonal difficulties, and challenges in adapting to the behavioral requirements of bariatric treatment [13,14]. Importantly, these findings should be interpreted as indicators of areas requiring targeted psychological assessment and support. Interventions aimed at improving emotion regulation, impulse control, coping with negative affects, and awareness of loss-of-control states might be especially useful in this subgroup. The present findings also support a broader conceptualization of FEDs in overweight or obesity. While eating-specific symptoms remain central to diagnosis, the psychological phenotype associated with FEDs appears to include transdiagnostic dimensions that cut across diagnostic boundaries. Motor impulsivity may represent the behavioral disinhibition component, whereas reduced anger control—pending replication given its preliminary status in this study—may tentatively represent an emotional-regulation component. Together, these dimensions may help explain why some individuals with overweight or obesity develop clinically significant eating-disorder psychopathology whereas others, despite comparable weight severity or body dissatisfaction, do not meet the criteria for FEDs [13,15,16]. Rather than representing only dysfunctional symptoms, these behaviors may also express an individual’s attempt to regulate tensions between conscious adaptation and emotionally charged aspects of the personality. While this interpretation extends beyond the empirical scope of the present study, it underscores the potential value of therapeutic approaches that foster emotional awareness, symbolic elaboration, and psychological integration. Several limitations of the present pilot study should be acknowledged. First, the study had a small sample size, and findings should thus be considered as exploratory. The regression model was intentionally parsimonious, but the findings require replication in larger samples. Given the exploratory nature of the study and the absence of prior effect-size estimates for this specific population, no a priori sample-size or power calculation was performed. We did not compute a post hoc power analysis, as retrospective power estimates are statistically uninformative and can be misleading. Instead, we report 95% confidence intervals throughout and emphasize estimation precision when interpreting effect sizes (Hedges’ g) and odds ratios. The relatively small and unevenly distributed subgroup sizes should be considered when interpreting the width of these intervals, and replication in larger, prospective, adequately powered samples is warranted. Although missing data were primarily attributable to participants’ non-completion of the diagnostic interview (Section 2.5), we cannot rule out that unmeasured factors related to symptom severity or motivation influenced whether participants completed the full assessment pathway; this represents a further source of potential selection bias inherent to real-world retrospective clinical data. Second, the cross-sectional design prevents causal inferences. It is not possible to determine whether impulsivity and poor anger control contribute to FED onset, result from chronic eating-related distress, or interact bidirectionally with eating-disorder symptoms. Third, predictors for the regression model were selected through a two-step procedure combining an a priori conceptual restriction to the impulsivity and anger-regulation domains with univariate significance testing within those domains; this data-dependent element of the selection process, together with the low events-per-predictor ratio, confirm that the resulting odds ratios should not be interpreted as robust evidence of independent, generalizable predictors. In addition, most psychological variables were assessed using self-report instruments, possibly being influenced by response biases, insight, and social desirability. Although FED diagnoses were based exclusively on SCID-5-CV criteria and clinical judgment, interviewers were not blinded to self-report questionnaire scores and no formal inter-rater reliability assessment was performed; while questionnaire scores were not used as diagnostic criteria, we cannot fully exclude some indirect influence of this lack of blinding on diagnostic judgment, which represents a further limitation of the present retrospective design. Finally, a FED diagnosis was treated as a binary outcome, whereas FEDs exist along a continuum and may include heterogeneous clinical presentations. This could be particularly relevant if we consider the heterogeneity of the sample, which could have also been influenced by the recruitment of patients from two different clinical settings. Data on psychiatric comorbidities, pharmacological treatment, and ongoing psychological therapy were not systematically recorded in the present study protocol; these variables could therefore not be examined as potential confounders, and their possible role cannot be excluded. Despite these limitations, this study has several strengths. It focused on a clinically relevant sample of adults with overweight or obesity recruited from specialized clinical services, used DSM-5-TR FED diagnosis rather than only dimensional eating symptoms, and examined multiple psychological domains within the same sample. Moreover, by excluding eating-specific scales from the final exploratory regression, the analysis reduced criterion overlap and highlighted transdiagnostic correlates of FED status. The use of a parsimonious STAXI-2 Anger Control Index was further supported by the extremely high correlation between the two STAXI-2 anger-control scales, reducing redundancy and model complexity.

## 5. Conclusions

Compared with those without FEDs, individuals with overweight or obesity and FEDs showed, as expected, higher eating-disorder psychopathology—a finding that partly reflects the diagnostic classification itself rather than an independent psychological difference—and, more informatively, higher motor impulsivity, which emerged as the most robust transdiagnostic correlate of FED status across all sensitivity analyses performed. These findings suggest that the assessment of individuals with overweight or obesity may benefit from attention directed towards behavioral impulsivity, alongside eating-specific symptoms. Given the exploratory nature and limited sample size of this pilot study, larger samples are needed to confirm these findings. Future longitudinal, adequately powered studies should examine whether these dimensions predict eating behavior, adherence, weight-loss trajectories, and psychological outcomes.

## Figures and Tables

**Table 1:** Demographic, clinical, and psychological characteristics of the participants.

Variable	Total Sample (N = 50)
Age (years)	46.77 (13.82)
BMI, kg/m^2^	40.23 (5.93)
Female sex, *n* (%)	38 (76.0)
Male sex, *n* (%)	12 (24.0)
No FED, *n* (%)	29 (58.0)
FED, *n* (%)	21 (42.0)
BES	16.74 (10.06)
BSQ	120.92 (40.19)
BULIT-R	71.16 (25.88)
BIS-11 Total	62.56 (11.38)
STAXI-2 Anger Control-Out	31.64 (11.38)
STAXI-2 Anger Control-In	32.58 (11.51)

Note. Continuous variables are reported as M (SD); categorical variables are reported as *n* (%). Age was available for 48 participants and BMI for 49.

**Table 2:** Group comparisons between participants with and without FEDs.

Variable	No FED (*n* = 29)		FED (*n* = 21)		*t*(df)	*p*	*g* [95% CI]
	M	SD	M	SD			
BES	12.03	8.03	23.24	9.00	−4.63 (48)	<0.001	1.31 [0.69, 1.92]
BSQ	112.14	39.59	133.05	38.71	−1.86 (48)	0.069	0.53 [−0.04, 1.09]
BULIT-R	60.17	23.68	86.33	20.95	−4.04 (48)	<0.001	1.14 [0.54, 1.74]
BIS-11 Total	58.24	8.65	68.52	12.18	−3.49 (48)	0.001	0.99 [0.40, 1.57]
BIS-11 Motor impulsivity	13.31	2.63	19.24	5.32	−4.71 (27.12)	<0.001	1.47 [0.84, 2.09]
BIS-11 Attentional impulsivity	19.69	3.75	21.71	4.00	−1.83 (48)	0.073	0.52 [−0.05, 1.08]
BIS-11 Non-planning impulsivity	24.83	4.74	27.90	5.92	−2.04 (48)	0.047	0.58 [0.01, 1.14]
BIS-11 Self-Control	12.93	3.21	14.14	3.73	−1.23 (48)	0.224	0.35 [−0.21, 0.90]
STAXI-2 State Anger	33.55	7.38	28.29	12.80	1.69 (29.55)	0.101	0.52 [−0.04, 1.08]
STAXI-2 Trait Anger	33.03	7.89	26.67	10.58	2.33 (35.29)	0.026	0.69 [0.12, 1.26]
STAXI-2 Trait Anger-Temperament	22.25	7.46	14.86	8.79	3.10 (39.01)	0.004	0.90 [0.32, 1.49]
STAXI-2 Trait Anger-Reaction	24.24	6.89	16.43	8.61	3.44 (37.12)	0.001	1.01 [0.42, 1.59]
STAXI-2 Anger Expression-Out	30.17	8.30	21.62	8.91	3.49 (48)	0.001	0.98 [0.42, 1.57]
STAXI-2 Anger Expression-In	34.10	6.34	26.33	8.60	3.68 (48)	<0.001	1.04 [0.45, 1.63]
STAXI-2 Anger Control-Out	36.76	8.18	24.57	11.54	4.14 (33.99)	<0.001	1.23 [0.63, 1.84]
STAXI-2 Anger Control-In	37.86	8.55	25.29	11.21	4.50 (48)	<0.001	1.27 [0.66, 1.88]

Note. BES = Binge Eating Scale; BMI = Body Mass Index; BSQ = Body Shape Questionnaire; BULIT-R = Bulimia Test-Revised; BIS-11 = Barratt Impulsiveness Scale-11; FED = Feeding and Eating Disorder; STAXI-2 = State-Trait Anger Expression Inventory, version 2. When Levene’s test was significant, Welch’s *t*-test was reported instead of Student’s *t*-test. For STAXI-2 Trait Anger-Temperament, one participant in the No FED group had a missing value (No FED *n* = 28 for this comparison only). STAXI-2 scores are T scores. *t* values refer to the No FED − FED difference; Hedges’ *g* is reported in absolute value.

**Table 3:** Exploratory logistic regression of variables associated with FED status.

Predictor	B	SE	Wald	OR	95% CI for OR	*p*
Motor impulsivity	0.388	0.157	6.139	1.474	[1.084, 2.003]	0.013
Non-planning impulsivity	−0.078	0.108	0.521	0.925	[0.748, 1.143]	0.470
STAXI-2 Anger Control Index	−0.084	0.041	4.152	0.920	[0.848, 0.997]	0.042

Note. Omnibus χ^2^(3) = 26.43, *p* < 0.001; Nagelkerke R^2^ = 0.552; Hosmer–Lemeshow χ^2^(8) = 4.51, *p* = 0.808. At the conventional 0.5 probability cutoff, overall classification accuracy was 78.0% (sensitivity = 71.4%, specificity = 82.8%, positive predictive value = 75.0%, negative predictive value = 80.0%); given the exploratory aim of the analysis and the absence of external or internal predictive validation, these classification measures should not be interpreted as evidence of predictive utility. The Anger Control Index is a study-specific composite (mean of Anger Control-Out and Anger Control-In) and is not part of standard STAXI-2 scoring.

**Table 4:** Comparison of recruitment sites on demographic, clinical, and psychological characteristics.

Variable	Pre-Bariatric Setting (*n* = 32)	Specialist Outpatient Center (*n* = 18)	Test Statistic	*p*	g [95% CI]
FED, *n* (%)	7 (21.9)	14 (77.8)	χ^2^(1) = 14.78	<0.001	–
Age, years, M (SD)	46.75 (10.66)	46.81 (19.06)	Welch t(19.83) = −0.01	0.990	0.00 [−0.59, 0.59]
Female sex, %	75.0	77.8	χ^2^(1) = 0.05	0.825	–
BMI, kg/m^2^, M (SD)	41.70 (5.14)	37.46 (6.46)	t(47) = 2.51	0.016	0.74 [0.14, 1.34]
BES, M (SD)	13.53 (8.84)	22.44 (9.76)	t(48) = −3.30	0.002	−0.96 [−1.56, −0.36]
BSQ, M (SD)	116.94 (37.51)	128.00 (44.81)	t(48) = −0.93	0.356	−0.27 [−0.84, 0.30]
BULIT-R, M (SD)	59.19 (16.59)	92.44 (26.06)	Welch t(24.93) = −4.89	<0.001	−1.60 [−2.25, −0.95]
BIS-11 Total, M (SD)	59.31 (9.69)	68.33 (12.13)	t(48) = −2.88	0.006	−0.84 [−1.43, −0.24]
BIS-11 Motor Impulsivity, M (SD)	13.84 (3.44)	19.28 (5.32)	Welch t(25.15) = −3.90	<0.001	−1.27 [−1.90, −0.65]
BIS-11 Attentional Impulsivity, M (SD)	20.38 (3.43)	20.83 (4.83)	Welch t(26.85) = −0.36	0.725	−0.11 [−0.68, 0.46]
BIS-11 Non-planning Impulsivity, M (SD)	24.91 (4.93)	28.28 (5.73)	t(48) = −2.19	0.034	−0.64 [−1.22, −0.05]
BIS-11 Self-Control, M (SD)	12.50 (3.01)	15.11 (3.64)	t(48) = −2.73	0.009	−0.79 [−1.38, −0.20]
STAXI-2 State Anger, M (SD)	36.06 (3.06)	22.94 (12.99)	Welch t(18.07) = 4.22	<0.001	1.59 [0.94, 2.24]
STAXI-2 Trait Anger, M (SD)	36.12 (4.03)	20.11 (7.74)	Welch t(22.30) = 8.18	<0.001	2.80 [2.00, 3.60]
STAXI-2 Anger Expression-Out, M (SD)	32.88 (4.40)	15.39 (4.09)	t(48) = 13.83	<0.001	4.01 [3.03, 4.99]
STAXI-2 Anger Expression-In, M (SD)	35.75 (4.93)	22.11 (5.12)	t(48) = 9.27	<0.001	2.69 [1.91, 3.47]
STAXI-2 Anger Control-Out, M (SD)	39.19 (4.27)	18.22 (6.51)	Welch t(25.40) = 12.26	<0.001	3.99 [3.01, 4.97]
STAXI-2 Anger Control-In, M (SD)	40.25 (4.57)	18.94 (5.99)	t(48) = 14.13	<0.001	4.10 [3.10, 5.10]

Note. BES = Binge Eating Scale; BMI = Body Mass Index; BSQ = Body Shape Questionnaire; BULIT-R = Bulimia Test-Revised; BIS-11 = Barratt Impulsiveness Scale-11; FED = Feeding and Eating Disorder; STAXI-2 = State-Trait Anger Expression Inventory, version 2. STAXI-2 scores are T scores. When Levene’s test was significant, Welch’s *t*-test was reported. Age was available for 48 participants (*n* = 32 pre-bariatric, *n* = 16 specialist center) and BMI for 49 (*n* = 32 and *n* = 17). Test statistics and *g* refer to the pre-bariatric minus specialist center difference.

## Data Availability

The datasets generated and analyzed during the current study are not publicly available due to privacy and ethical restrictions.

## Supplementary Materials

The following supporting information can be downloaded at: https://www.mdpi.com/article/10.3390/jcm15208156/s1, Table S1. Normality (Shapiro-Wilk) and outlier screening for all sixteen Table 2 outcomes, by FED group. Table S2. Mann-Whitney U sensitivity analysis for all sixteen group comparisons in Table 2. Table S3.1. Robustness of the multivariable model: original (MLE) vs. Firth bias-corrected logistic regression. Table S3.2. Standardized odds ratios (per 1-SD increment) for the original multivariable model. Table S3.3. Sensitivity models entering Anger Control-Out and Anger Control-In separately (instead of the combined index).
