# Supplementary material for: Impulsivity and Anger Regulation in Adults with Overweight or Obesity and Feeding and Eating Disorders: An Exploratory Cross-Sectional Study
Source: J Clin Med. 2026 Oct 20;15(20):8156. doi: 10.3390/jcm15208156 (PMC13706579; doi:10.3390/jcm15208156)
Supplement: Supplementary file 1 [file jcm-15-08156-s001.zip › jcm-4504137-supplementary.pdf]

Table S1. Normality (Shapiro-Wilk) and outlier screening for all sixteen Table 2 outcomes, by FED group.

Variable	Shapiro-Wilk No FED W (p)	Shapiro-Wilk FED W (p)	Outliers (Tukey 1.5×IQR)
BES	0.953 (.218)	0.980 (.923)	None
BSQ	0.963 (.394)	0.959 (.493)	1 (FED, low value)
BULIT-R	0.864 (.001)	0.972 (.778)	3 (No FED, high values); 1 (FED, high value)
BIS-11 Total	0.979 (.820)	0.981 (.943)	1 (No FED, low value)
BIS-11 Motor Impulsivity	0.969 (.535)	0.973 (.807)	3 (No FED, high values)
BIS-11 Attentional Impulsivity	0.974 (.671)	0.898 (.032)	None
BIS-11 Non-planning Impulsivity	0.979 (.808)	0.965 (.611)	1 (FED, high value)
BIS-11 Self-Control	0.945 (.139)	0.973 (.790)	None
STAXI-2 State Anger	0.751 (<.001)	0.885 (.018)	8 (No FED, low values)
STAXI-2 Trait Anger	0.845 (.001)	0.905 (.044)	3 (No FED, low values)
STAXI-2 Trait Anger-Temperament	0.633 (<.001)	0.851 (.004)	4 (No FED, low values)
STAXI-2 Trait Anger-Reaction	0.707 (<.001)	0.890 (.022)	4 (No FED, low values)
STAXI-2 Anger Expression-Out	0.883 (.004)	0.845 (.003)	5 (No FED, low values)
STAXI-2 Anger Expression-In	0.964 (.407)	0.959 (.501)	1 (No FED, low value)
STAXI-2 Anger Control-Out	0.866 (.002)	0.949 (.333)	2 (No FED, low values)
STAXI-2 Anger Control-In	0.857 (.001)	0.952 (.367)	6 (No FED, low values)

Note. W = Shapiro-Wilk statistic; outliers identified using Tukey's 1.5× interquartile range rule within each group.

Table S2. Mann-Whitney U sensitivity analysis for all sixteen group comparisons in Table 2.

**Variable**	**U**	**p (unadjusted)**	**FDR-adjusted p[1]**	**Rank-biserial r**
BES	501.0	<.001	.001	-0.645
BSQ	387.0	.107	.114	-0.271
BULIT-R	502.0	<.001	.001	-0.649
BIS-11 Total	454.5	.003	.007	-0.493
BIS-11 Motor Impulsivity	510.5	<.001	.001	-0.677
BIS-11 Attentional Impulsivity	404.5	.049	.072	-0.328
BIS-11 Non-planning Impulsivity	401.0	.058	.078	-0.317
BIS-11 Self-Control	364.5	.240	.240	-0.197
STAXI-2 State Anger	221.5	.102	.114	0.273
STAXI-2 Trait Anger	212.5	.072	.088	0.302
STAXI-2 Trait Anger-Temperament	168.5	.011	.018	0.427
STAXI-2 Trait Anger-Reaction	161.5	.005	.009	0.470
STAXI-2 Anger Expression-Out	160.5	.005	.009	0.473
STAXI-2 Anger Expression-In	149.5	.002	.006	0.509
STAXI-2 Anger Control-Out	124.0	<.001	.001	0.593
STAXI-2 Anger Control-In	117.0	<.001	.001	0.616

[1] *Benjamini-Hochberg FDR correction applied across all sixteen comparisons.*

Table S3.1. Robustness of the multivariable model: original (MLE) vs. Firth bias-corrected logistic regression.

Predictor	Original MLE OR [95% CI]	p	Firth bias-corrected OR [95% CI]	p
Motor impulsivity	1.474 [1.084, 2.003]	.013	1.380 [1.052, 1.810]	.020
Non-planning impulsivity	0.925 [0.748, 1.143]	.470	0.940 [0.774, 1.142]	.534
Anger Control Index	0.920 [0.848, 0.997]	.042	0.929 [0.861, 1.003]	.059

Note. Firth's penalized (bias-reduced) logistic regression; confidence intervals are Wald-type, based on the penalized model's information matrix.

Table S3.2. Standardized odds ratios (per 1-SD increment) for the original multivariable model.

Predictor	Standardized OR (per 1-SD) [95% CI]	p
Motor impulsivity (SD = 4.92)	6.749 [1.490, 30.561]	.013
Non-planning impulsivity (SD = 5.43)	0.655 [0.208, 2.066]	.470
Anger Control Index (SD = 11.31)	0.388 [0.156, 0.965]	.042

Table S3.3. Sensitivity models entering Anger Control-Out and Anger Control-In separately (instead of the combined index).

Model variant	Anger-related predictor OR [95% CI]	p	Motor impulsivity OR [95% CI]	p
Anger Control-Out only	0.922 [0.852, 0.998]	.045	1.476 [1.089, 2.001]	.012
Anger Control-In only	0.922 [0.851, 0.998]	.044	1.474 [1.084, 2.004]	.013

Note. Each row is a separate logistic regression including Motor impulsivity, Non-planning impulsivity, and the single anger-control subscale indicated. Anger Control-Out and Anger Control-In were not entered together due to severe multicollinearity (r = .953; VIF > 11).
